# Differential effects of SARS-CoV-2 variants on central nervous system cells and blood–brain barrier functions

**DOI:** 10.1186/s12974-023-02861-3

**Published:** 2023-08-03

**Authors:** Alizé Proust, Christophe J. Queval, Ruth Harvey, Lorin Adams, Michael Bennett, Robert J. Wilkinson

**Affiliations:** 1 Tuberculosis Laboratory, The Francis Crick Institute, London, NW1 1AT UK; 2 High Throughput Screening Laboratory, The Francis Crick Institute, London, NW1 1AT UK; 3 Worldwide Influenza Centre, The Francis Crick Institute, London, NW1 1AT UK; 4 Department of Infectious Diseases, Imperial College London, London, W12 0NN UK; 5 Institute of Infectious Disease and Molecular Medicine and Department of Medicine, Wellcome Centre for Infectious Diseases Research in Africa, University of Cape Town, Observatory, Cape Town, 7925 Republic of South Africa

**Keywords:** SARS-CoV-2, Blood–brain barrier, Central nervous system, Brain, Excitotoxicity, Astrocytes, Brain microvascular endothelial cells, Microglia, Pericytes

## Abstract

**Background:**

Although mainly causing a respiratory syndrome, numerous neurological symptoms have been identified following of SARS-CoV-2 infection. However, how the virus affects the brain and how the mutations carried by the different variants modulate those neurological symptoms remain unclear.

**Methods:**

We used primary human pericytes, foetal astrocytes, endothelial cells and a microglial cell line to investigate the effect of several SARS-CoV-2 variants of concern or interest on their functional activities. Cells and a 3D blood–brain barrier model were infected with the wild-type form of SARS-CoV-2, Alpha, Beta, Delta, Eta, or Omicron (BA.1) variants at various MOI. Cells and supernatant were used to evaluate cell susceptibility to the virus using a microscopic assay as well as effects of infection on (i) cell metabolic activity using a colorimetric MTS assay; (ii) viral cytopathogenicity using the xCELLigence system; (iii) extracellular glutamate concentration by fluorometric assay; and (iv) modulation of blood–brain barrier permeability.

**Results:**

We demonstrate that productive infection of brain cells is SARS-CoV-2 variant dependent and that all the variants induce stress to CNS cells. The wild-type virus was cytopathic to all cell types except astrocytes, whilst Alpha and Beta variants were only cytopathic for pericytes, and the Omicron variant cytopathic for endothelial cells and pericytes. Lastly wild-type virus increases blood–brain barrier permeability and all variants, except Beta, modulate extracellular glutamate concentration, which can lead to excitotoxicity or altered neurotransmission.

**Conclusions:**

These results suggest that SARS-CoV-2 is neurotropic, with deleterious consequences for the blood–brain barrier integrity and central nervous system cells, which could underlie neurological disorders following SARS-CoV-2 infection.

**Supplementary Information:**

The online version contains supplementary material available at 10.1186/s12974-023-02861-3.

## Introduction

Discovered in Wuhan in December 2019, the severe acute respiratory syndrome coronavirus 2 (SARS-CoV-2) has caused over six million deaths and 520 million confirmed cases of Coronavirus Disease 2019 (COVID-19) over the two first years of the pandemic [1]. Five coronaviruses pathogenic for humans (HCoV) are described as neuroinvasive (SARS-CoV-2, MERS-CoV, SARS-CoV, HCoV-OC43 and HCoV-229E), but their ability to enter the central nervous system (CNS) is not well characterised [2–4]. HCoV can invade the CNS via two potential routes: (i) the olfactory pathway by which viruses reach the olfactory bulb via the olfactory nerves and further spread in the CNS and (ii) a hematogenous pathway involving either a leakage of the blood–brain barrier (BBB, paracellular transmigration), or a passage of virions through cells of the BBB (transcellular migration) [5].

Although brain microvascular endothelial cells are the principal component of the BBB, the ultimate control of its barrier phenotype resides with pericytes and astrocytes. In addition, microglia and perivascular macrophages interact with the BBB and mediate immunity at the barrier. Every constituent cell type contributes to the BBB's integrity and any dysfunction could potentially culminate in critical pathophysiology, such as neuroinflammation or even neurodegeneration [6]. Neurological symptoms have been reported in patients infected by neuroinvasive HCoV from mild symptoms such as headaches, confusion, seizures to more severe complications and neurological disorders including acute disseminated encephalomyelitis, multiple sclerosis, Parkinson’s disease, neuromuscular disorders, and Guillain–Barre syndrome [7–17].

A systematic review and meta-analysis of 27 studies showed that COVID-19 patients in their acute phase of infection and up to 7 months after infection had cognitive deficits associated with COVID-19 (including, processing speed, executive function, phonemic and category fluencies, memory encoding and recall) compared to healthy controls [18]. The longitudinal study from Douaud and colleagues showed that even a mild infection by SARS-CoV-2 could shrink the brain (up to 2% of brain volume loss) and thin the grey matter compared to healthy people. They also correlated the loss of grey matter to the mental decline measured (processing speed, attention, and visual screening ability) [19]. The cellular basis of these long-lasting impairments remains unclear.

The aims of this study were to evaluate the overall effects of SARS-CoV-2 in CNS cells and investigate possible mechanisms behind the neurological decline. Considering the rapid mutation of SARS-CoV-2 and its impact on viral transmissibility and/or severity, we also investigated whether SARS-CoV-2 mutations modulate effects of the infection on the CNS.

For this purpose, we infected astrocytes, pericytes, endothelial cells and microglia and an in vitro BBB model with the WT (Wild Type, Wuhan strain) form of SARS-CoV-2 as well as 5 variants of concern or interest carrying specific mutations modulating their infectivity and transmissibility including Alpha, Beta, Delta, Eta, and Omicron (Additional file 1) and analysed their neurotropism and effects on CNS cells and BBB function. We showed that susceptibility and permissivity to the viruses was variant dependent and that they all induced stress in CNS cells, the outcome of which depended on the variant and cell type. Also, using an in vitro BBB model, we demonstrated that WT virus increases BBB permeability and that all variants, except Beta, modulate extracellular glutamate levels.

Taken together, these results demonstrate an important deleterious impact of SARS-CoV-2 infection on both CNS cells and the BBB and support the clinical evidence describing neurological disorders related to SARS-CoV-2 infection.

## Methods

### Commercial human cells and culture conditions

Human cerebral microvascular endothelial cells** (**hCMEC/d3, Merck Life Science) were grown on collagen I-coated (150 µg/mL, Sigma-Aldrich) flasks/wells using the EndoGRO™-MV complete Media Kit supplemented with 1 ng/mL FGF-2 (“hCMEC/d3 medium”, both from Merck Life Science). Human microglial cells (HMC3, ATCC) were grown in EMEM (ATCC) supplemented with 10% heat-inactivated foetal bovine serum (Corning). Cell lines were used at a passage between 3 and 12 throughout. Human Brain Vascular Pericytes (HBVP, ScienCell) were grown on poly-L-lysine (2 µg/cm^2^, Sigma-Aldrich) coated flasks in complete Pericyte Medium (ScienCell). All cells were grown at 37 °C in the presence of 5% CO_2_.

### Primary human astrocyte isolation

Human foetal astrocytes were isolated from foetal brain samples as previously described [20, 21]. Briefly, blood vessels and meninges were removed from the foetal brain tissue (15 to 20 gestational weeks). Thereafter, the tissue was minced, treated with 0.2 mg/ml DNase I (Sigma-Aldrich) and 0.25% trypsin (ThermoFisher Scientific) for 30 min before being passed through a 70-µm cell strainer (Corning). The flow-through was plated in petri dishes for adherent cells (Sarstedt) at a final concentration of 6–8 × 10^7^ cells/petri in MEM supplemented with 10% FBS, 100 U/ml penicillin, 100 µg/ml streptomycin, 0.3 mg/ml L-glutamine, 1 mM sodium pyruvate, 1X MEM nonessential amino acids, 0.5 µg/ml amphotericin B, and 2.5 ml of glucose solution (all from ThermoFisher Scientific). Astrocytes were grown at 37 °C (5% CO_2_) and left undisturbed for 2 weeks, thereafter they were passaged once every week. To ensure cell purity, all experiments were conducted using cells on the third to six passages.

### Viral strains

The SARS-CoV-2 reference isolate (referred to as ‘Wild-type’, GISAID accession ID: EPI_ISL_407073) was obtained from the Respiratory Virus Unit, Public Health England (PHE), UK. The Alpha isolate (lineage B.1.1.7, GISAID accession ID: EPI_ISL_693401) was obtained from PHE, through Prof. Wendy Barclay (Imperial College London) through the Genotype-to-Phenotype National Virology Consortium (G2P-UK). The Beta isolate (lineage B.1.351) was kindly provided by Prof. Alex Sigal and Prof. Tulio de Oliveira. However, sequencing of viral isolates identified the Q677H and R682W mutations at the furin cleavage site in approximately 50% of the genomes, which was maintained upon passage in cell culture. The Delta isolate (lineage B.1.617.2, GISAID accession ID: EPI_ISL_1731019) and the Eta isolate (lineage B.1.525) were kindly provided by Prof. Wendy Barclay (Imperial College London) through the G2P-UK. The Omicron isolate (lineage BA.1) was kindly provided by Prof. Gavin Screaton (University of Oxford) through the G2P-UK.

### Virus production

All viral isolates were expanded in African green monkey kidney cells (VERO, subtype V1) as previously described [22]. Briefly, cells were washed once with DMEM (Sigma) supplemented with 1% FBS (Biosera) to wash off the V1 growing media (DMEM + 10% FBS). Then, where the titre of the virus inoculum was known, 100 pfu/ml in DMEM supplemented with 1% FBS was added to each flask and incubated at room temperature for 30 min. Some infections were done directly from original material which had no titre, in which case the inoculum was titrated from a 10^–1^ to 10^–5^ dilution and the resulting virus was assessed for titre. Flasks were then topped up with DMEM supplemented with 1% FBS. Cells were incubated at 37 °C (5% CO_2_) for approximatively 4 days until extensive cytopathic effects were observed. Supernatant was harvested and clarified by centrifugation at 2000 rpm for 10 min then aliquoted and frozen at − 80 °C. All procedures related to virus culture were conducted in a biosafety level 3 + (BSL3 +), according to Health and Safety Executive (HSE) guidelines. All virus stocks generated were sequence-validated prior to use.

### Detection of ACE2, TMPRSS2, CD147 and NRP1 expression by microscopy

CNS cells were cultured on µ-slide 8-well ibiTreat (Ibidi) for 2 to 3 days. Cells were then fixed in 2% paraformaldehyde for 30 min at 4 °C before permeabilisation and blocking with a buffer containing 1X PBS (pH 7.4), 1% bovine serum albumin (BSA, Sigma), 30% normal goat serum (Abcam) and 0.2% Triton X-100 (VWR Chemicals) for 30 min at room temperature. Cells were then incubated for 2 h at room temperature with an anti-ACE2 monoclonal antibody (ThermoFisher Scientific), an anti-TMPRSS2 polyclonal antibody (ThermoFisher Scientific), an anti-CD147 monoclonal antibody (R&D System), and/or an anti-NRP1 monoclonal antibody (ThermoFisher Scientific), all at a dilution of 1:50. Slides were then stained either with Alexa Fluor 647 goat anti-rabbit secondary antibody (ACE2 and TMPRSS2) and/or Alexa Fluor 488 goat anti-rat secondary antibody (NRP1 and CD147), both at a dilution of 1:500 (ThermoFisher Scientific). Cells were then labelled with NucBlue™ Fixed Cell ReadyProbes (ThermoFisher Scientific) following the manufacturer’s instruction to stain nuclei. Images were acquired using a Zeiss Invert880 with Airyscan microscope (Zeiss) with a 40 × objective and analysed with Zen 3.5 Blue Edition Software (Zeiss).

### CNS cell susceptibility to SARS-CoV-2

CNS susceptibility to SARS-CoV-2 was assessed using a microscopic assay previously described [23] with some modifications. Briefly, cells were seeded in clear bottom, black 96-well plates (Greiner bio-one) at 2 × 10^4^ cells/well and cultured for 24 h before virus infection. Cells were then incubated with SARS-CoV-2 isolates (WT, Alpha, Beta, Delta, Eta, or Omicron) at various MOI (0.1, 1, 2.5, 5) for 24 h before being washed thoroughly with PBS to remove unbound virus. Cells were then fixed with 4% formaldehyde (Thermo Fisher Scientific) for 1 h after 24 h (day of the wash), 2 days, 4 days, or 6 days of infection. Cells were subsequently permeabilised with 0.2% TritonX-100 and 3% BSA in PBS and stained for SARS-CoV-2 N protein with Alexa488-labelled-CR3009 antibody produced in-house and cellular DNA using DAPI (Sigma-Aldrich) [24]. Whole-well imaging at 5 × magnification was acquired using an Opera Phenix (Perkin Elmer) and fluorescent areas calculated using Harmony v4.9 software (Perkin Elmer).

### CNS cell infection with SARS-CoV-2

CNS cells seeded either on inserts (blood–brain barrier (BBB) model, described later), E-plates (xCELLigence) or 96-well plates (MTS assay) were infected with SARS-CoV-2 isolates (WT, Alpha, Beta, Delta, Eta or Omicron) at two MOI (0.1 and 1) for up to 6 days. MTS, BBB permeability and glutamate assays were performed after 2, 4 and 6 days of infection. For the xCELLigence assay, the impedance was continuously recorded for 6 days after infection.

### MTS assay

The metabolic activity of CNS cells was assessed using CellTiter 96™ AQueous Nonradioactive Cell Proliferation Assay (conversion of the tetrazolium salt MTS [3-(4,5-dimethylthiazol-2-yl)-5-(3-carboxymethoxyphenyl)-2-(4-sulfophenyl)-2H-tetrazolium)] to a purple formazan in the presence of phenazine ethosulfate) following the manufacturer’s instructions (Promega). Absorbance at 490 nm was measured using a Synergy 2 multi-mode microplate reader (Biotek instruments).

### xCELLigence assay

The xCELLigence Real Time Cell Analysis Dual Purpose system (RTCA DP, Agilent) allows the continuous and non-invasive recording of electrode impedance integrated into the bottom of E-plates 16. The number, size and shape of cells affect the impedance which is reported using the unitless parameter cell index (CI). The CI is directly proportional to the relative change in electrical impedance normalised by the background value. Recording of CI values and analysis were performed using the Real Time Cell Analysis Software Pro version 2.3 (Agilent). In brief, hCMEC/d3 cells, astrocytes, HMC3 and HBVP were evenly distributed in 16-well E plates pre-coated with Collagen-I (hCMEC/d3 cells) or poly-L-lysine (astrocytes and HBVP) in three to four replicates for each condition. To prevent edge effects, we allowed the cells to attach to the 16-well E-plates at room temperature for 30 min before being inserted in the RTCA DP system, inside an incubator (37 °C, 5% CO2) for continuous impedance recording. The hCMEC/d3 cells reached their confluent phase and formed a tight barrier on day 4–5. Astrocytes, HMC3 and HBVP were confluent on the day following their seeding. Cells were then infected by SARS-CoV-2 as described previously. The uninfected condition was used as baseline and negative control in our analysis.

### Tight and adherens junction expression

hCMEC/d3 cells were seeded on 12-well plates pre-coated with Collagen-I for 6 days before infection with SARS-CoV-2 isolates (WT, Alpha, Beta, Delta, Eta or Omicron) at two MOI (0.1 and 1) for up to 6 days. Total RNA was extracted after 2, 4 and 6 days of infection using the NucleoSpin RNA Kit (Macherey-Nagel) according to the manufacturer’s instructions and reverse transcribed to cDNA with M-MLV RT Polymerase (Promega). Tight and adherens junctions’ expression was assessed by SYBR-Green Quantitative RT-PCR using QuantStudio™ 7 Flex Real-Time PCR System (Thermo Fisher Scientific) following the manufacturer’s instructions. Primer (used at 0.3 μM) sequences were forward 5′-AGAAGGATGTTTATCGTCGCATT-3′ and reverse 5′-CCAAGAGCCCAGTTTTCCAT-3′ for TJP1, forward 5′-CAAGTCGAGAGGAAACTGTTG-3′ and reverse 5′-TCTGACTTCAGGTTCAGAAGAG-3′ for F11R, forward 5′-GTCCAATATTTTGTGGGACAAGG-3′ and reverse 5′-GGCACGTCCTGTGTGCCT -3′ for OCLN, forward 5′-GTGCTCTACCTGTTTTGCG-3′ and reverse 5′-GACGGGTCGTAAAACTCG-3′ for CLDN5, forward 5′- CAGCCCAAAGTGTGTGAGAA -3′ and reverse 5′- CGGTCAAACTGCCCATACTT-3′ for CDH5, and forward 5′-TAGAGGGACAAGTGGCGTTC-3′ and reverse 5′-CGCTGAGCCAGTCAGTGT-3′ for 18S. Amplification of target genes was normalised to the geometric mean of 18S ribosomal cDNA. A standard curve was drawn for each gene of interest using serial dilutions of pooled cDNA from all samples.

### In vitro human BBB model system

We used a co-cultivation model including hCMEC/d3 cells and a mix of pericytes and astrocytes seeded on each side of a porous insert allowing cell–cell contacts as previously described [25, 26]. Microglia were seeded on the bottom of the well to mimic their localisation in the CNS. Briefly, cell culture inserts for 24-well plates with a 3.0-μm pore size, translucent PET membrane (Corning Life Sciences) were coated with 150 μg/mL collagen-1 (Sigma-Aldrich) on the upper side and with poly-L-lysine (2 µg/cm^2^, Sigma-Aldrich) on the basal side. A mix of HBVP (10^4^/insert) and astrocytes (5 × 10^4^/insert) were then seeded on the basal side of the membrane and incubated for 4 h before hCMEC/d3 cells (2.5 × 10^4^/insert) were seeded on the upper side of the membrane. Cells were allowed to grow in 150 μL (upper chamber) and 750 μL (collector) of BBB medium 1, composed of 50% hCMEC/d3 medium and 50% HBVP medium, for 7 days to reach confluence (medium in both upper chamber and collector were refreshed with 150 μL and 750 μL of BBB medium 1, respectively, at day 4). HCM3 (5 × 10^3^/well) were then seeded at the bottom of the well containing the inserts and media changed (upper chamber and collector) to a mix of 50% hCMEC/d3 medium, 25% HBVP medium and 25% HMC3 medium (BBB medium).

BBB integrity was assessed by measuring permeability to dextran-rhodamine B. Briefly, the culture medium in the upper chamber was replaced with BBB medium supplemented with 0.5 mg/mL 70-kDa dextran-rhodamine B (Life Technologies). After 5 h, the fluorescence intensity in the collector was measured using a Synergy 2 multi-mode microplate reader (Biotek). Samples displaying dextran-rhodamine B permeability greater than 20% of the empty control insert were discarded.

### BBB permeability assay

The effect of SARS-CoV-2 infection on BBB integrity was assessed by measuring the permeability of the BBB to dextran-rhodamine B. After 2, 4 or 6 days of infection, the culture medium in the insert was replaced with BBB medium supplemented with 0.5 mg/mL 70-kDa dextran-rhodamine B. The fluorescence intensity in the collector was measured using a Synergy 2 multi-mode microplate reader (Biotek) after 5 h.

### Glutamate assay

Glutamate quantification was monitored using the Glutamate assay kit (Abcam) on cell experiment collectors following the manufacturer’s instructions. Optical density at 450 nm was measured using a Synergy 2 multi-mode microplate reader (Biotek instruments).

### Statistical analysis

**Fig. 1:**
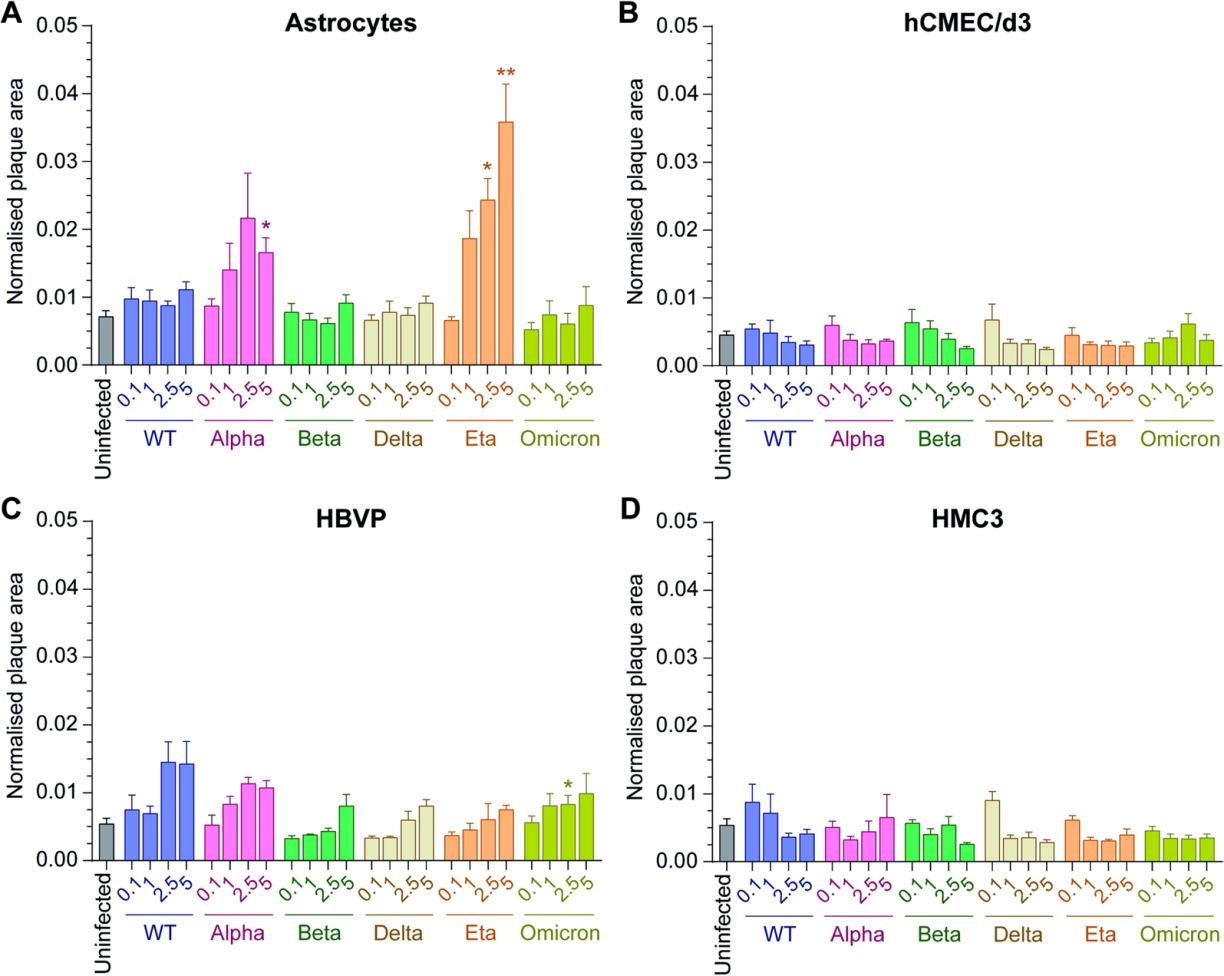
Variation in susceptibility of CNS cells to SARS-CoV-2. **A** Astrocytes, **B** hCMEC/d3, **C** HBVP and **D** HMC3 were infected for 24 h at various MOI (0.1 – 1 – 2.5 – 5) with WT (blue), Alpha (pink), Beta (green), Delta (brown), Eta (orange) or Omicron (yellow-green) viruses. The uninfected condition of each CNS cell (grey) is shown and form the background. The mean and SEM for 3 different donors/passages are shown. The normalised plaque area (fluorescent viral areas (µm^2^) normalised over fluorescent nuclei areas (µm^2^)) is presented. Asterisks denote statistically significant data as determined by two-way analysis of variance (ANOVA) with corrections for multiple comparisons (Dunnett) (*P < 0.05, **P < 0.01)

**Fig. 2:**
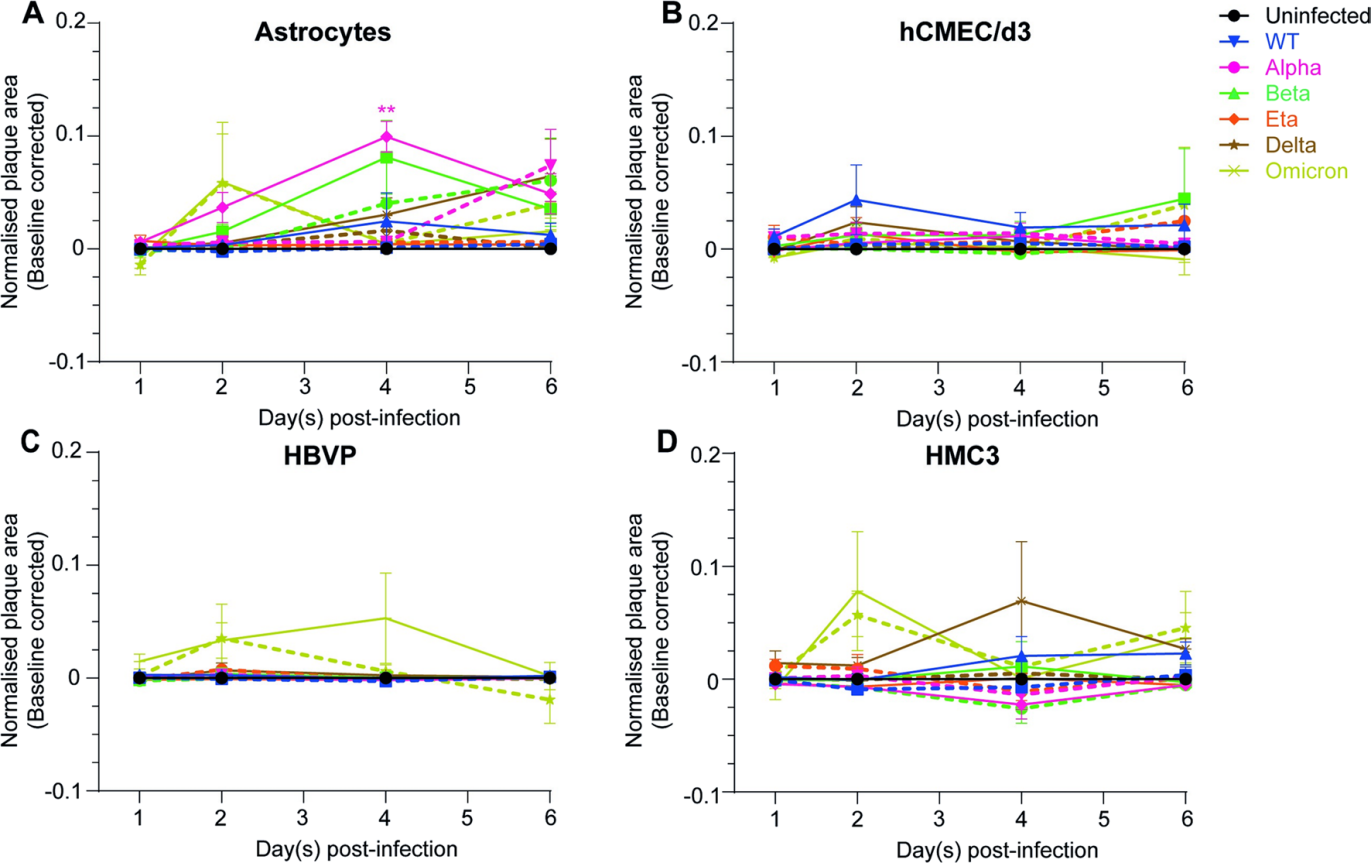
Productive infection of CNS cells is SARS-CoV-2 variant dependent. **A** Astrocytes, **B** hCMEC/d3, **C** HBVP and **D** HMC3 were incubated with WT (blue), Alpha (pink), Beta (green), Delta (brown), Eta (orange) or Omicron (yellow-green) viruses at MOI 0.1 (dashed lines) or MOI 1 (full lines) for 24 h, washed to removed unbound viruses and stained after 2, 4 or 6 days of infection. The uninfected condition is represented with a black full line. The mean and SEM for 3 different donors/passages are shown. Fluorescent viral areas (µm^2^) have been normalised over fluorescent nuclei areas (µm^2^) and the baseline (uninfected control) removed. Asterisks denote statistically significant data as determined by two-way analysis of variance (ANOVA) with corrections for multiple comparisons (Dunnett) (*P < 0.05, **P < 0.01)

**Fig. 3:**
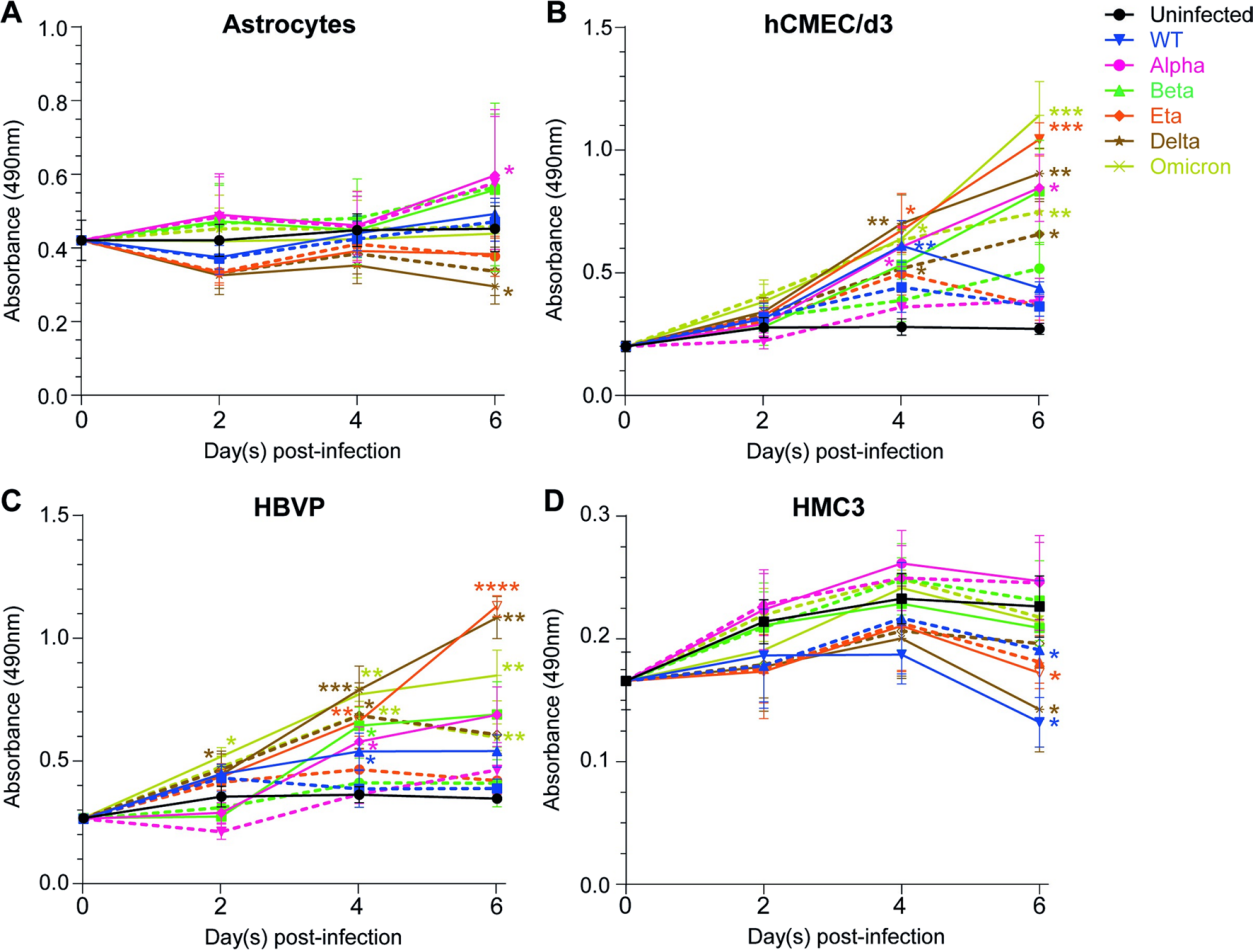
SARS-CoV-2 infection modulates the metabolic activity of CNS cells. The metabolic activity of **A** astrocytes, **B** hCMEC/d3, **C** HBVP and **D** HMC3 was assessed using an MTS assay following 2, 4 or 6 days of infection at MOI 1 (full lines) or MOI 0.1 (dashed lines) with WT (blue), Alpha (pink), Beta (green), Delta (brown), Eta (orange) or Omicron (yellow-green) viruses. The uninfected condition is represented with a black line. Results are presented in raw data (i.e. absorbance read at 490 nm). The mean and SEM for 4 to 5 different donors/passages are shown. Asterisks denote statistically significant data as determined by one-way analysis of variance (ANOVA) with corrections for multiple comparisons (Tukey) (*P < 0.05, **P < 0.01, ***P < 0.001, ****P < 0.0001)

**Fig. 4:**
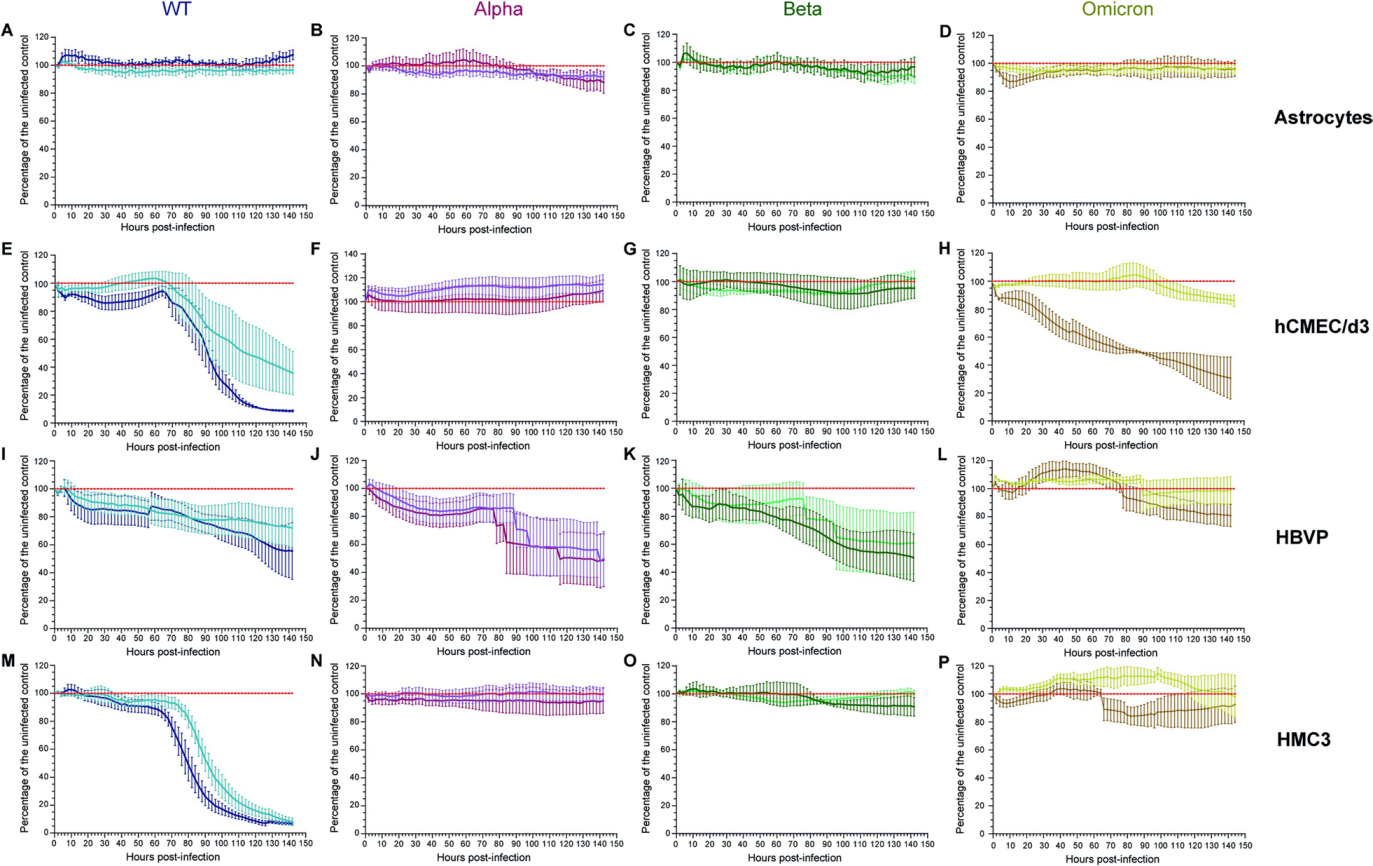
SARS-CoV-2 cytopathic effect on CNS cells. **A**–**D** Astrocytes, **E**–**H** hCMEC/d3, **I**–**L** HBVP and **M**–***P*** HMC3 were infected with WT (**A**, **E**, **I**, **M**), Alpha (**B**, **F**, **J**, **N**), Beta (**C**, **G**, **K**, **O**) and Omicron (**D**, **H**, **L**, **P**) viruses at MOI 0.1 (WT: light blue; Alpha: purple; Beta: light green; Omicron: yellow) or MOI 1 (WT: dark blue; Alpha: burgundy; Beta: dark green; Omicron: brown) for 6 days and the impedance was recorded in real time continuously. The mean and SEM for 3 to 5 different donors/passages are presented in percentage of the uninfected control (red)

**Fig. 5:**
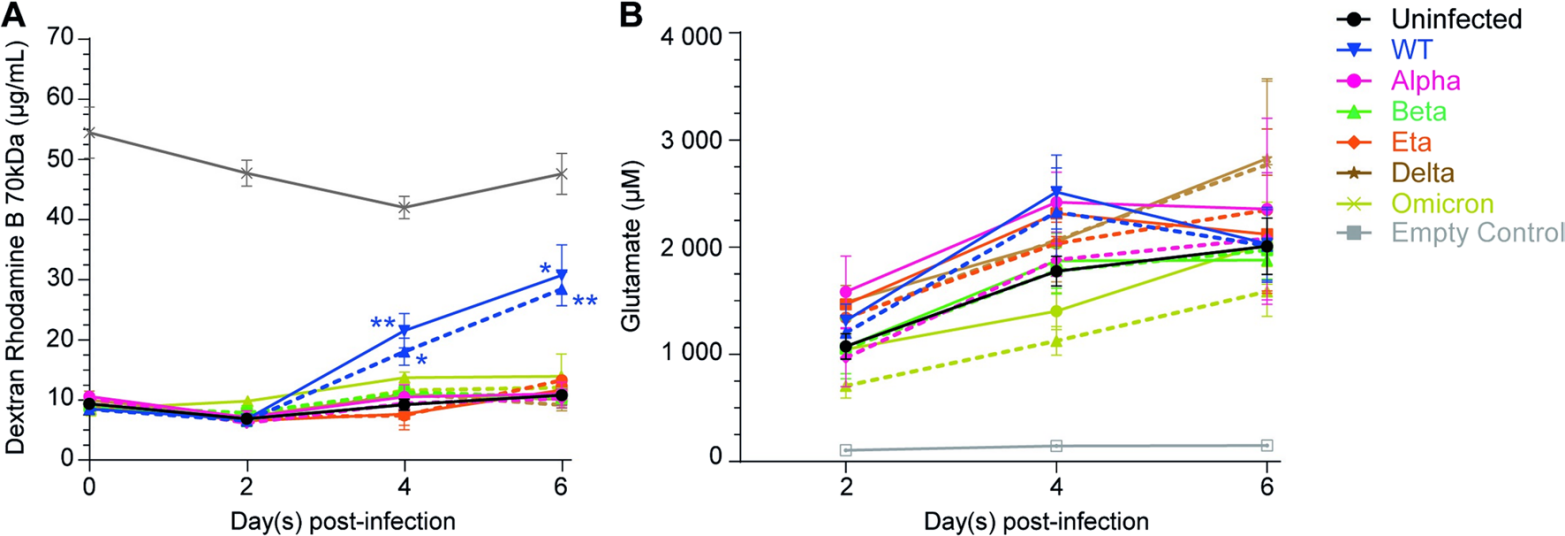
WT virus increases BBB permeability while WT, Alpha, Delta, Eta and Omicron infections modulate extracellular glutamate. BBB were infected at MOI 1 (full lines) or MOI 0.1 (dashed lines) with WT (blue), Alpha (pink), Beta (green), Delta (brown), Eta (orange) or Omicron (yellow-green) viruses and we measured **A** BBB permeability (dextran-rhodamine assay) and **B** extracellular glutamate at 2, 4 or 6 days post-infection. An empty control insert (grey) was used to estimate the passive diffusion of dextran-rhodamine from the upper chamber to the collector and the concentration of glutamate in the BBB medium over time. Data are presented in raw data (i.e. concentration of Dextran-rhodamine B 70 kDa in µg/mL or concentration of glutamate in µM). The mean and SEM for 3 to 4 different experiments are shown. Asterisks denote statistically significant data as defined by one-way analysis of variance (ANOVA) with corrections for multiple comparisons (Tukey) (*P < 0.05, **P < 0.01)

**Fig. 6:**
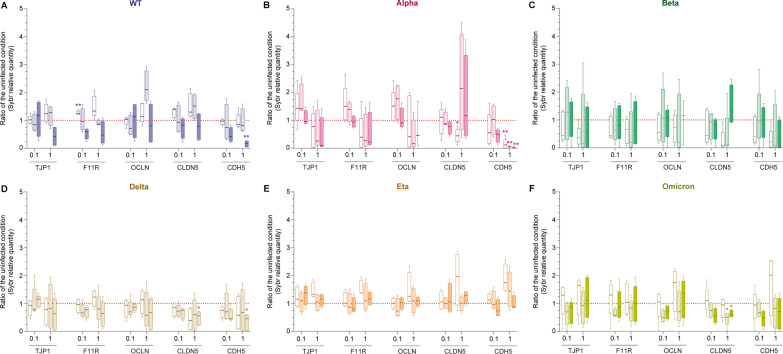
SARS-CoV-2 impact on endothelial cell tight junction and adherens junction gene expression. Endothelial cell gene expression of TJP1 (also known as ZO-1), F11R (encoding JAM-A), OCLN (Occludin), CLDN5 (Claudin-5) and CDH5 (encoding VE-Cadherin) was assessed by SYBR-Green Quantitative RT-PCR following 2, 4 or 6 days of infection at MOI 1 (labelled “1”) or MOI 0.1 (labelled “0.1”) with **A** WT, **B** Alpha, **C** Beta, **D** Delta, **E** Eta or **F** Omicron viruses. Results for 3 to 5 different donors/passages are presented in ratio of the uninfected condition (dashed red line) in box and whiskers plots (median and min to max). Asterisks denote statistically significant data as defined by two-way analysis of variance (ANOVA) with corrections for multiple comparisons (Dunnett) (*P < 0.05, **P < 0.01)

Statistical analysis was performed using GraphPad Prism version 9.3.1. Statistical details of each experiment (statistical tests used, exact value of n, dispersion, and precision measures) can be found in the figure legends. Mean ± SEM are used throughout Figs. 1, 2, 3, 4 and 5 while for Fig. 6 box and whiskers plots with median ± range are used. Statistical analyses were performed on raw data for all experiments. A threshold *P* value of ≤ 0.05 (*) was considered statistically significant. When non-statistically significant, no star or “ns” have been added in figures for the sake of clarity.

## Results

### Expression of ACE2, TMPRS22, CD147 and NRP1 entry factors by CNS cells

SARS-CoV-2 entry to the cells requires the presence of ACE2 (entry receptor) and TMPRSS2 (primes the SARS-CoV-2 spike protein) [27, 28]. CD147, also known as basigin (BSG) or extracellular matrix metalloproteinase inducer (EMMPRIN), has also been proposed as an alternative receptor to SARS-CoV-2 [29]. NRP1 (stabilises viral binding to the cell) binding to the virus is not essential but facilitates SARS-CoV-2 entry and infectivity [30]. We thus assessed whether the cells used in our study expressed those entry factors.

Additional file 2 shows that astrocytes, endothelial cells (hCMEC/d3), pericytes (HBVP) and microglia (HMC3) expressed NRP1 and CD147. On the contrary, the SARS-CoV-2 receptor ACE2 was only expressed by astrocytes and microglia and weakly by endothelial cells. Finally, the serine protease TMPRSS2 was expressed by all cell types, although the level of expression was lower in pericytes.

These results suggest that astrocytes and microglia express ACE2, TMPRSS2, CD147 and NRP1 which indicates potential SARS-CoV-2 entry in those cells.

### Susceptibility of CNS cells to SARS-CoV-2 after 24 h of incubation with the virus

Though the expression of ACE2 and TMPRSS2 was low in some CNS cells, we next investigated whether those cells could be infected by the WT virus and 5 variants, Alpha, Beta, Delta, Eta and Omicron, at different multiplicities of infection (MOI) of 0.1, 1, 2.5, and 5. Cells and viruses were incubated for 24 h to ensure any potential cell infection, whilst keeping the cells alive, even at high MOI.

As a control we showed the infectivity of our 6 viral strains in VERO E6 cells line, an established model for SARS-CoV-2 infection. As shown in Additional file 3, all 6 viral variants used in the study infected VERO E6 cells at all MOI tested.

We then applied the same conditions to determine the susceptibility of the CNS cells, i.e. astrocytes, microglia (HMC3), endothelial cells (hCMEC/d3) and pericyte (HBVP), to SARS-CoV-2.

Figure 1 shows that astrocytes (Fig. 1A) were only infected with Alpha and Eta variants, reaching a maximum normalised plaque area of 0.022 ± 0.007 at MOI 2.5 for the Alpha variant and 0.036 ± 0.006 for the Eta variant compared to the background 0.007 ± 0.001. Pericytes (Fig. 1C) were infected by all variants from MOI 1 (Alpha, maximum normalised plaque area at MOI 2.5 and 5: 0.011 ± 0.001 and Omicron, maximum normalised plaque area at MOI 5: 0.01 ± 0.003), MOI 2.5 (WT, maximum normalised plaque area at MOI 2.5: 0.015 ± 0.003) or MOI 5 (Beta, maximum normalised plaque area: 0.008 ± 0.002; Delta and Eta, maximum normalised plaque area: 0.008 ± 0.001). The extent of infection was, however, not great compared to the background (0.005 ± 0.001) and, though apparent, did not reach statistical significance in some cases.

Compared to VERO E6 cells (S2 Fig), SARS-CoV-2 infection of astrocytes and pericytes was much less pronounced. The maximum normalised plaque areas reached for VERO E6 cells infections were 0.797 ± 0.056 (WT, MOI 5), 0.649 ± 0.178 (Alpha, MOI 1), 0.492 ± 0.153 (Beta, MOI 5), 0.569 ± 0.01 (Delta, MOI 5), 0.669 ± 0.031 (Eta, MOI 5) and 0.057 ± 0.014 (Omicron, MOI 5), with VERO E6 cell background being as low as that of CNS cells (0.008 ± 0.002).

Finally, endothelial cells (Fig. 1B) and microglia (Fig. 1D) seemed only infected at MOI 0.1 with the Delta and Beta variants (endothelial cells, maximum plaque areas: 0.007 ± 0.002 and 0.006 ± 0.002 for Delta and Beta, respectively) or WT and Delta variants (microglia, maximum plaque areas: 0.009 ± 0.003 and 0.009 ± 0.001 for WT and Delta, respectively) but no clear dose effect were observed for any variant. Also, and similarly, to astrocytes and pericytes, these infections were very low as endothelial cells and microglia backgrounds were 0.005 ± 0.001 and 0.005 ± 0.001, respectively. Together, these data suggest that only astrocytes and pericytes are susceptible to SARS-CoV-2.

### Viral replication within CNS cells is SARS-CoV-2 variant dependent

We then assessed whether the CNS cells were productively infected by SARS-CoV-2 or if the infection was abortive. Cells were incubated with the 2 lowest MOI previously tested (0.1 and 1) for 24 h and washed to remove unbound viral particles. The cells were then left undisturbed for up to 6 days post-infection and the viral replication was ascertained by fluorescence microscopy.

Figure 2A shows that astrocytes reached a peak of infection at day 2 with Omicron at both MOI (MOI 0.1: 0.058 ± 0.044; MOI 1: 0.059 ± 0.053) and at day 4 with Alpha (0.099 ± 0.016) and Beta (0.081 ± 0.033) variants at MOI 1. Productive infection of astrocytes by Alpha and Beta variants was MOI dependent as the infection increased steadily until the end of the experiment, 6 days post-infection, at MOI 0.1 (Alpha: 0.074 ± 0.034, Beta: 0.061 ± 0.039). Astrocytes were also productively infected by the Delta variant at MOI 1 whose infection increased continuously until 6 days post-infection (0.064 ± 0.034). Normalised plaque areas of endothelial cells infected with WT (MOI 1, 0.044 ± 0.031), Beta (MOI 1, 0.045 ± 0.044) and Omicron (MOI 1, 0.039 ± 0.051) viruses were slightly increased after 2 (WT) and 6 (Beta and Omicron) days of infection and indicated replication of those variants within endothelial cells (Fig. 2B). Figure 2C shows no variation of pericyte normalised plaque areas for all variants except Omicron, though not statistically significant (maximum normalised plaque area at day 2 for MOI 0.1: 0.035 ± 0.030 and day 4 for MOI 1: 0.054 ± 0.040), suggesting that pericytes were only productively infected by the Omicron variant. Finally, microglia were productively infected by Omicron at both MOI with a peak of infection at day 2 (MOI 0.1: 0.057 ± 0.019; MOI 1: 0.078 ± 0.053). Microglia were also infected by WT and Delta variants at a MOI of 1, reaching a peak of infection at day 4 (0.069 ± 0.052) for the Delta variant and increasing continuously until 6 dpi (0.023 ± 0.01) for the WT virus (Fig. 2D).

We concluded that (i) Eta infection was abortive while Omicron infection was productive in all the CNS cells tested; (ii) infection of pericytes by all SARS-COV-2 variants except Omicron was abortive; (iii) astrocytes were productively infected by WT, Alpha, Beta, Delta and Omicron variants; (iv) WT, Beta and Omicron infection in endothelial cells resulted in viral replication; (vi) microglia were productively infected by WT, Delta and Omicron variants.

### SARS-CoV-2 infection modulates metabolic activity of CNS cells

Despite being sparsely infected by SARS-CoV-2; CNS cells may react to the presence of the virus or infected cells nearby. Therefore, we measured the effect of SARS-CoV-2 infection on CNS cell mitochondrial metabolic activity, a marker of viability as only viable living cells with intact mitochondria and cell membrane can catalyse the reaction (6 variants, 2 MOI: 0.1 and 1).

Figure 3A shows that metabolic activity of astrocytes was not modulated by WT and Omicron infection but increased up to 1.32-fold and 1.25-fold after 6 days of infection with Alpha and Beta variants, respectively. By contrast, the metabolic activity of astrocytes was decreased by up to 1.53- and 1.19-fold after 6 days of infection with Delta and Eta variants, respectively. The metabolic activity of microglia was slightly and transiently increased in response to Alpha variant infection, reaching its peak at day 4 (increase up to 1.14-fold). WT, Delta and Eta infection had a negative impact on metabolic activity of microglia, reducing it by up to 1.71-, 1.58- and 1.31-fold, respectively (Fig. 3D). Endothelial cells (Fig. 3B) and pericytes (Fig. 3C) showed the same profile. Indeed, the metabolic activities of both cell types were increased by all 6 SARS-CoV-2 variants tested in a MOI-dependent manner (the higher the MOI, the higher the metabolic activity). Metabolic activity in response to WT infection peaked at 4 days and increased up to 2.18-fold, for endothelial cells and up to 1.49-fold for pericytes. Alpha, Beta, Delta, Eta, and Omicron infections induced a continuous increase of endothelial cell and pericyte metabolic activity, reaching an increase up to 3.12-fold, 3.07-fold, 3.33-fold, 3.85-fold, and 4.21-fold, respectively, for endothelial cells and 1.98-fold, 1.99-fold, 3.13-fold, 3.2-fold and 2.45-fold, respectively, for pericytes.

These results suggest that the 6 variants of SARS-CoV-2 induced transient or continuous stress to endothelial cells and pericytes. Also, WT (only for microglia), Delta and Eta infections caused stress to astrocytes and microglia. Whether this stress impacts cell viability and induces cell death was not established.

### WT and Omicron variant disrupt the endothelial barrier

We next monitored in real time the effect of SARS-CoV-2 infection on astrocytes, pericytes, microglial monolayers as well as on cerebral microvascular endothelial barrier integrity using the impedance-based xCELLigence RTCA system. This system has previously been shown to efficiently monitor tight junction integrity in human brain endothelial cells [31, 32].

Figure 4A–D shows that astrocyte monolayers were not disrupted by WT, Alpha, Beta and Omicron infection at MOI 0.1 and 1. Alpha and Beta variants had no effect on microglial monolayers (Fig. 4N–O) and endothelial barrier integrity (Fig. 4F, G) but disrupted pericyte monolayers with a decrease of 51.3 ± 19% (Alpha, MOI 1), 50.3 ± 19.6% (Alpha, MOI 0.1), 49.6 ± 16.9% (Beta, MOI 1) and 39.2 ± 22% (Beta, MOI 0.1) in the percentage of cells compared to the uninfected control (Fig. 4J, K). The WT virus negatively affected the endothelial barrier as well as pericytes and microglial monolayers in a MOI-dependent manner (Figs. 4E, I, M, respectively). The percentage of cells after 6 days of infection was decreased to 92.7 ± 1.7% (hCMEC/d3, MOI 1), 61.6 ± 14.7% (hCMEC/d3, MOI 0.1), 44.47 ± 20.3% (HBVP, MOI 1), 27.9 ± 13.9% (HBVP, MOI 0.1), 94.9 ± 1.6% (HMC3, MOI 1) and 93.6 ± 1.9% (HMC3, MOI 0.1) compared to the uninfected control. Finally, the Omicron variant disrupted the endothelial barrier (4H) and pericyte monolayer at MOI 1 (4L) with a decrease up to 14 ± 4.2% (hCMEC/d3, MOI 0.1), 69.4 ± 15.1% (hCMEC/d3, MOI 1) and 19.3 ± 7.9% (HBVP, MOI 1) in the percentage of cells compared to the uninfected control after 6 days of infection. Both Delta and Eta infections had no effect on the resistance in CNS cell monolayers (Additional file 4).

These results indicate that SARS-CoV-2 did not exert cytopathic effect on astrocytes. The WT virus was cytopathic for pericytes and microglia and disrupted the endothelial barrier. The Omicron variant was only cytopathic for pericytes (MOI 1) and endothelial cells. Finally, Alpha and Beta variants were also cytopathic for pericytes.

### WT virus infection increases BBB permeability

The reduction of endothelial barrier integrity induced by the WT and Omicron viruses led us to investigate the impact of SARS-CoV-2 on a 3D human BBB model. The impact of the infections on BBB permeability was assessed by measuring the diffusion of dextran-rhodamine B (70 kDa) from the upper chamber to the collector after 2, 4 or 6 days of infection. An empty control insert with no cells was used to assess passive diffusion of the fluorescent marker and mimicked a BBB with 100% permeability (Fig. 5A, grey line).

Consistent with the breakdown of endothelial monolayer resistance previously described, infection with the WT virus significantly increased BBB permeability reaching 43.5 ± 6.4% (MOI 0.1) and 51.0 ± 4.7% (MOI 1) permeability after 4 days of infection and 62.4 ± 5.5% (MOI 0.1) and 66.7 ± 9.1% (MOI 1) permeability 6 days post-infection (Fig. 5A). Compared to the uninfected BBB (15.6 ± 1.0% at day 2, 23.0 ± 1.5% at day 4 and 24.7 ± 1.7% at day 6), these increases correspond to a 1.9-fold and 2.2-fold increase (MOI 0.1 and 1, respectively) at day 4 and a 2.5- and 2.7-fold increase (MOI 0.1 and 1, respectively) at day 6. However, and despite the significant impact of Omicron infection on the endothelial barrier integrity, the Omicron variant at MOI 1 only slightly increased BBB permeability in our 3D BBB model, reaching 21.0 ± 1.3% (day 2), 27.7 ± 1.6% (day 4) and 28.0 ± 6.3% (day 2) of permeability (1.3-, 1.2- and 1.1-fold increase at 2, 4 and 6 days post-infection, respectively, compared to the uninfected BBB). These data partially confirm those of Fig. 4, indicating that infection with the WT virus has greatest effect on BBB permeability.

### Downregulation of tight and adherens junction proteins expression is SARS-CoV-2 variant dependant

We next investigated the endothelial cell gene expression of tight junction proteins (ZO-1, JAM-A, Occludin and Claudin-5) and adherens junction protein (VE-Cadherin) in response to 2, 4 or 6 days of infection with SARS-CoV-2.

Figure 6A shows that the WT virus strongly decreased the expression of the tight junction proteins TJP1 (MOI 1, up to 57.4% median decrease at 6dpi) and JAM-A (MOI 0.1 and 1, up to 46.4% and 46.2% median decrease at 6dpi, respectively) as well as the expression of the adherens junction VE-Cadherin (MOI 0.1 and MOI 1, up to 57.5% and 82.9% median decrease at 6dpi, respectively). Interestingly, expression of TJP1, F11R, OCLN and CLDN5 were transiently increased after 2 or 4 days of infection before their downregulation (median increase up to 28.4% for TJP1, 33.5% for F11R, 110.4% for OCLN and 51.6% for CLDN5). While not being cytopathic for endothelial cells, Alpha variants strongly downregulated TJP1, F11R, OCLN and CDH5 expression (Fig. 6B, median decrease for TJP1: up to 89.1%; F11R: up to 77.6%; OCLN: up to 83.2%; CDH5: up to 97.8%). Remarkably, CLDN5 expression was firstly decreased (median decrease of 55.2% at 2dpi, MOI 1) before increasing (up to 114% median increase at 4dpi, MOI 1). Figure 6C shows that all the genes tested were transiently but strongly decreased following 2 to 4 days of infection with the Beta variant (median decrease of TJP1: up to 83.6%; F11R: up to 82.1%; OCLN: up to 82.3%; CLDN5: up to 91.1% and CDH5: up to 93.3%) before a more moderate downregulation (TJP1, F11R, OCLN and CDH5) or upregulation (CLDN5) after 6 days of infection. By contrast, Delta variant induced a sustainable downregulation of all genes (Fig. 6D; median decrease for TJP1: up to 37%; F11R: up to 36.6%; OCLN: up to 42.6%; CLDN5: up to 60.9% and CDH5: up to 54.7%). While not strongly impacting tight junction expression at low MOI, Eta variant at MOI 0.1 induced 26.5% median decrease of CDH5 expression after 6 days of infection (Fig. 6E). Eta variant at MOI 1 also transiently induced an increase in tight and adherens junction expression at 2 dpi (median increase for TJP1: 33.5%; F11R: 38.3%; OCLN: 25.9%; CLDN5: 96.6% and CDH5: 74.5%) before expression levels returned to the uninfected condition ones after 6 days of infection. Finally, the Omicron variant induced a sustained downregulation of CLDN5 and CDH5 expression (Fig. 6F; median decrease for CLDN5: up to 49.6%; CDH5: up to 50.1%). F11R and OCLN genes were transiently upregulated (median increase for F11R: up to 30.6%; OCLN: up to 75.3%) after 2 days on infection followed by a transient decrease at 4 dpi (median decrease for F11R: up to 42.2%; OCLN: up to 51.2%) and a return to the uninfected control levels expression at 6 days post-infection.

### SARS-CoV-2 infection modulates extracellular glutamate concentration

The concentration of the neurotransmitter glutamate in the extracellular space is tightly regulated to prevent excitotoxicity. This homeostasis is mainly maintained by astrocytes which take up and release glutamate [33]. Considering that (i) astrocytes support the integrity of the BBB, (ii) astrocytes are productively infected by some SARS-CoV-2 variants and (iii) the metabolic activity of astrocytes is affected by SARS-CoV-2 infection, we therefore investigated the effect of SARS-CoV-2 on extracellular glutamate in our BBB model. Extracellular glutamate was measured in the collector after 2, 4 or 6 days of infection. An empty control insert with no cells was used to assess the concentration of glutamate in the BBB medium over time (Fig. 5B, grey line).

Figure 5B shows that WT, Alpha, and Eta variants at high MOI transiently increased extracellular glutamate, with a peak at day 4. At that time point, the concentrations of glutamate were increased by up to 1.3-fold ± (+ 739 µM) for WT, 1.4-fold (+ 644 µM) for Alpha and 1.3-fold (+ 539 µM) for Eta compared to the uninfected BBB (black line). Infection with the Delta variant continuously increased extracellular glutamate until day 6 and reached an increase of 1.4-fold (+ 819 µM) at an MOI of 1. Interestingly, Omicron infection induced a decrease in extracellular glutamate until reaching the uninfected condition at day 6 at MOI 1. Decreases of 1.5-fold (-368 µM, MOI 0.1) at day2, 1.6-fold (-650 µM, MOI 0.1) and 1.3-fold (-372 µM, MOI 1) at day4 and 1.3-fold (-421 µM, MOI 0.1) at day 6 were observed.

## Discussion

Due to the impact of the COVID-19 pandemic on worldwide health and economy in the last 3 years, many have studied the pathogenesis of SARS-CoV-2, mainly in the lungs or in pulmonary cells. Despite brain manifestations in infected patients and the accumulation of evidence of cognitive impairment, very little is known on the mechanism(s) by which the viral particles access the CNS and their impact on brain cells and homeostasis. In the current study, we sought to investigate SARS-CoV-2 brain tropism and evaluate the impact of infection on BBB and CNS cell metabolism. Most importantly, with only published one study evaluating the risk of neurological and psychiatric outcomes after Alpha, Delta or Omicron infection [34], we also investigated the impact of SARS-CoV-2 genomic mutations on neuropathogenesis by comparing the original Wuhan strain with 5 variants of concern or interest, Alpha, Beta, Delta, Eta and Omicron.

SARS-CoV-2 uses ACE2 and TMPRSS2 to enter the cells [27, 28]. Their expression by brain cells is still controversial, some researchers reporting that endothelial cells, pericytes and/or glial cells highly express ACE2 and/or TMPRSS2 while others suggest the opposite [35–40]. Whether those cells express ACE2 and TMPRSS2 seems to depend on the brain region, the cells used (primary cells, cell lines), the way the primary cells have been isolated, the age of the brain (foetal or adult), the species or even the way the expression has been measured. There is a paucity of data regarding NRP1 expression by human CNS cells, Zhang and colleagues showed that astrocytes, especially mature, brain endothelial cells and microglia/macrophages expressed NRP1 [40]. As for ACE2 and TMPRSS2, NRP1 expression is also brain region dependent [41]. By contrast, it is widely accepted that CD147 is expressed in the human brain, including on astrocytes, pericytes, endothelial cells and microglia [38, 42]. We have demonstrated in this study that primary foetal astrocytes isolated by adherence, commercial primary pericytes (HBVP), a human microglial cell line (HMC3) and the widely used hCMEC/d3 cell line express NRP1, CD147 and TMPRSS2, although the expression of the latter was weak in pericytes. Also, the ACE2 entry receptor is expressed by astrocytes, microglia and weakly by endothelial cell but pericytes do not express ACE2.

From our investigations, summarised in Table 1, we observed different tropisms among cell types and variants. Although infected at a high MOI by WT, Alpha, Beta, Delta and Eta variants, only Omicron replicated in pericytes. Considering that no ACE2 has been detected on pericytes, it would suggest that SARS-CoV-2 uses another receptor to enter pericytes. Using HEK-293 T cells (no ACE2 and NRP1 expression) transfected with a NRP1 plasmid, it has been shown that neuropilin-1 alone slightly promotes SARS-CoV-2 infection of these cells [30]. Thus, SARS-CoV-2 may enter pericytes using NRP1.

**Table 1:** Summary of SARS-CoV-2 effects on the BBB and CNS cells

		WT	Alpha	Beta	Delta	Eta	Omicron
*Astrocytes*							
Infection		Non-infected/abortive	Productive	Productive	Productive	Abortive	Productive
Metabolic activity		=	+	+	−	−	=
Cytopathic		No	No	No	No	No	No
*Endothelial cells*							
Infection		Productive	Non-infected /abortive	Productive	Non-infected /abortive	Non-infected / abortive	Productive
Metabolic activity		+	+	+	+	+	+
Cytopathic		Yes	No	No	No	No	Yes
Tight and adherens junctions expression at 6dpi	TJP1	−	−	−	−	=	=
	F11R	−	−	−	−	=	=
	OCLN	=	−	=	−	=	=
	CLDN5	=	+	+	−	=	−
	CDH5	−	−	−	−	=	−
*Pericytes*							
Infection		Abortive	Abortive	Abortive	Abortive	Abortive	Productive
Metabolic activity		+	+	+	+	+	+
Cytopathic		Yes	Yes	Yes	No	No	Yes
*Microglia*							
Infection		Productive	Non-infected / abortive	Non-infected / abortive	Productive	Non-infected / abortive	Productive
Metabolic activity		−	+	=	−	−	=
Cytopathic		Yes	No	No	No	No	No
*3D BBB model*							
Permeability		+	=	=	=	=	±
Glutamate levels		+	+	=	+	+	-

“Infection” (Figs. 1 and 2). Productive: cells are infected productively by the virus (Figs. 1 and 2: infection detected and increased). Abortive: cells are infected by the virus (Fig. 1: infection detected) but no replication (Fig. 2: no infection detected). Non-infected /abortive: cells are either not infected, or infection is not detected because too low (Fig. 1: no infection detected) and no infection is detected in Fig. 2

“Metabolic activity”, “Tight and adherens junctions expression”, “Permeability” and “Glutamate levels” (Figs. 3 and 5). = : no change. + : increase. -: decrease. ± : slight increase

“Cytopathic” (Fig. 4). Yes: virus is cytopathic for the cells. No: virus is not cytopathic for the cells

Crunfli and colleagues have recently showed that SARS-CoV-2 infects and replicates in human brain astrocytes of COVID-19 patients [43] which corroborate our findings on astrocytes. However, in our study, productive infection of CNS cells by most variants appeared transient, reaching a peak of infection at 2- or 4-days post-infection. One possibility is that the virions produced are non-infectious and failed to re-infect the cells.

Finally, CNS cells were either not infected by the Eta variant or their infection was abortive. Thus, WT, Alpha, Beta, Delta, and Omicron variants were the only variants that led to productive infection of CNS cells. Infections with Alpha, Beta, Delta, and Omicron variants were higher than infection with the WT virus on those cells. Their only common mutation not shared with the Eta variant is in the non-structural protein 12 (nsp12, RNA-dependent RNA polymerase) on the ORF1b (P314L), responsible for the control of genome replication [44], and thus this may impact viral replication and production in the brain. Similarly, it is possible that the mutation P314F in ORF1b of the Eta variant could impact its replication due to a change of fitness. Our data from microglia and endothelial cells also confirm previous results on productive infection of those cells by the WT virus [45, 46] but highlights how the mutations can affect the infectivity and pathogenicity between the different variants. Indeed, both studies used the WT virus (EPI_ISL_407193 and EPI_ISL_402119, respectively) and stated that microglia and endothelial cells were infected by SARS-CoV-2, which, we demonstrate, is confirmed only for WT, Beta and Omicron variants (endothelial cells) or WT, Delta and Omicron variants (microglia). In line with this, Kase and colleagues very recently compared the infectivity of WT, Delta and Omicron variants on iPSC-derived neurons, astrocytes and microglia [47]. Similarly to us, they showed an infection of microglia by all the tested variants. However, they saw no infection of astrocytes which is discordant from our work, as well as previous in vitro and in vivo studies [42, 43, 48]. As mentioned in the Kase study, this difference may be due to the method used to generate the astrocytes.

The MTS assay is sometimes interpreted to quantify cell death and proliferation, although it actually measures mitochondrial activity and allows the detection of cell stress when exposed to a pathogen or substance, in absence of direct cell death [49]. It has been proposed that SARS-CoV-2 hijacks host mitochondria and could induce mitochondrial dysfunction, potentially resulting in increased ROS production and reduced energy [50, 51] and we confirmed here that WT, Alpha, Beta, Delta, Eta and Omicron variants impact CNS cell metabolic activity. The WT, Delta and Eta variants decrease astrocyte (only Eta and Delta) and microglial metabolic activity and suggest reduced mitochondrial activity. By contrast, Alpha and Beta variants caused a slight increase in mitochondrial activity of astrocytes and microglia (Alpha only) which implies cell stress. There is no mutation only shared by Alpha and Beta variants (not shared with Delta, Eta and Omicron variants), thus multiple mutations must be implicated in microglial and astrocytes stress by both variants. Interestingly, all variants induced an increase in endothelial cell and pericyte mitochondrial activity, either transient (WT) or continuous (Alpha, Beta, Delta, Eta and Omicron variant), suggesting stress of those cells upon exposure to SARS-CoV-2. This supports Khaddai-Mallat and colleagues’ results showing that the S protein triggers oxidative and nitrosative stress in pericytes [52]. Such an increase in mitochondrial activity is concordant with a hyper-inflammatory phase and suggests a high degree of mitochondrial stress [53]. If prolonged, this mitochondrial stress may lead to cell death. In our study, the prolonged increase of mitochondrial activity upon Alpha, Beta, Delta, Eta and Omicron variants infection suggests that cellular redox buffers, like the ascorbate and glutathione, did not manage to restore redox level to normal and failed to protect mitochondrial homeostasis [54]. It is thus possible that the pro-oxidant and antioxidant systems have been dysregulated and reactive species overproduced which can lead to oxidative damage, functional dysregulation and, ultimately, cell death [55].

The conventional assay considered gold standard to study cytopathic affect rely on the measure of crystal violet or neutral red staining (CPE assays). However, theses assays have limitations with respect to their sensitivity and time resolution. CPE assays are also labour intensive and allow only endpoint data. The xCELLigence RTCA technology records in real-time the electrochemical impedance and provides an extremely sensitive readout of cell number. It also efficiently monitors tight junction integrity in brain endothelial cells [32]. We showed that Eta and Delta variants do not impair the impedance of any CNS cells implying no cytopathic effect of both variants on those cells. This suggests that the stress observed previously on CNS cells in response to Delta and Eta variants does not affect their viability. On the contrary, WT virus disrupts the endothelial barrier and is cytopathic for microglia and pericytes, implying excessive and potentially lethal stress to those cells. Again, Beta and Alpha virus exert the same profile by being both cytopathic for pericytes only. However, it indicates that endothelial cells and microglia remain viable despite being stressed for multiple days. The Omicron variant only negatively impacted endothelial barrier integrity and pericyte monolayer which is concordant with MTS assay data, for which only pericytes and endothelial cells were stressed by Omicron infection. Overall, our data suggest that pericyte viability is highly affected by the stress caused by SARS-CoV-2 exposure, compared to other CNS cells.

Despite the function of the BBB to restrict ingress of circulating microorganisms, it has been suggested that SARS-CoV-2 could infiltrate the BBB as cell-free viral particles, either by a transcellular pathway (through cells, with or without replication in endothelial cells), or a paracellular passive diffusion across a more permeable BBB (between cells) [56]. The WT and Omicron variant negatively affected the impedance of endothelial cell and pericyte monolayers seeming to confirm the latter hypothesis. We also corroborated it using an in vitro BBB model in which the WT virus, and to a lesser extent, Omicron variant, disrupt BBB integrity while the other variants tested had no effect. An increase of BBB permeability could lead to lymphocyte, monocyte and neutrophil migration into the brain which could induce neuroinflammation, ROS production, microglial and astroglial activation, oligodendrocyte dysfunction or myelin damage, thereby contributing to neurological disorder [57]. On the contrary, and although also using the WT virus, Zhang and colleagues did not observe any increase in BBB permeability in response to SARS-CoV-2 infection and proposed that the virus crosses the BBB via a transcellular pathway with disruption of the basement membrane mediated by MMP-9 [46]. However, our results show a delayed effect of infection, with BBB permeability increased after 2 days of infection, while Zhang et al. infected their BBB and cells for a maximum of 2 days, which may not have been long enough for an effect on the BBB and CNS cells. Yet, a transcellular pathway or even Trojan horse pathway (diapedesis of infected immune cells), not studied to date for SARS-CoV-2, cannot be excluded.

Tight and adherens junctions play a crucial roles in maintaining the integrity and selective permeability of the BBB [58]. Apart from the Eta variant, all SARS-CoV-2 variants tested in our study negatively impacted one or more tight (ZO-1, claudin-5, occludin and JAM-A) or adherens (VE-cadherin) junction protein expression. VE-cadherin was the most affected junctional protein, being strongly decreased by WT, Alpha and Delta variants. This contradicts the findings of Zhang and colleagues who found no impairment of occludin, ZO-1 and claudin-5 expression following SARS-CoV-2 infection [46]. This discrepancy could be explained by the difference in study model (K18-hACE2 mice and hamsters) as tight junction protein expressions greatly differ depending of the endothelial cell types [59].

Interestingly, WT, Alpha, Eta and Omicron variants showed transient increases in the expression of some junctional proteins in the early stages of infection, indicating an initial response by host cells to reinforce the barrier. However, sustained downregulation of these proteins has been observed at later time of infection suggesting viruses ability to overcome host defence. The observation that Alpha, Beta, Delta and Omicron variants impacted one or more tight and adherens junction protein expression but did not affect BBB permeability suggests a complex interplay between junctional protein expression and BBB functionality. While the downregulation of tight junctions and adherens junctions can contribute to compromised barrier integrity, compensatory mechanisms (involvement of PECAM-1 and nectins adhesion molecules on endothelial junctions assembly and localisation [60, 61]), cell signalling pathways (PKC and Rho GTPases signalling [62]) and the presence of other cells supporting the BBB (astrocytes and pericytes) may influence the overall permeability response.

Glutamate is the major excitatory neurotransmitter in the mammalian CNS and its concentration in the brain is tightly regulated, with both too little and too much glutamate being harmful, both can lead to defects in neurotransmission (too little glutamate) and excitotoxicity (too much glutamate) [63]. Excitotoxicity, reported to be involved in neurodegenerative disorders in humans [64], can occur acutely when mediated by the increase of extracellular glutamate levels. We demonstrated that, with the exception of the Beta variant, all SARS-CoV-2 variants tested either decrease (Omicron) or increase extracellular glutamate concentration, continuously (Delta) or transiently (WT, Alpha, Eta) compared to the uninfected control. Regulation of glutamate levels requires glutamate uptake which is mainly mediated by astrocytes via the glutamate transporter 1 (GLT-1). Our previous results showing infection of astrocytes and a modulation of their metabolic activity upon exposure to SARS-CoV-2 could explain this increase of glutamate levels. Two studies using another human coronavirus, HCoV-OC43 with a single mutation on the spike protein, has indeed reported a dysregulation of glutamate homeostasis and a significant downregulation of GLT-1 on astrocytes of infected mice astrocytes which was associated with motor dysfunctions [65, 66].

## Conclusions

Altogether, our work suggests that SARS-CoV-2 affects the normal physiological functions of the BBB and its cellular components and thus contributes to the wide spectrum of neurological manifestations of SARS-CoV-2 that have been observed clinically. More specifically, our results demonstrate that the WT virus and the Omicron variant may have a higher potential for neurological damage due to their ability to induce CNS cell stress, affect extracellular glutamate concentration, and damage BBB cellular components. The Eta, Delta, Beta, and Alpha variants seem to have comparatively lower impact on neurological health, as they either do not cause direct cellular death, do not affect glutamate concentration significantly, or do not induce BBB breakdown.

### Supplementary Information

**Additional file 1:** Mutations and impact on transmissibility and severity of Alpha, Beta, Delta, Eta and Omicron variants. ORF: Open Reading Frame; E: Envelope; N: Nucleocapsid; M: Matrix. ^1^: in-house sequencing; ^2^: covariants.org enable by GISAID; 3: European Centre for Disease Prevention and Control. Increased: evidence demonstrating an increased transmissibility/severity compared to the WT; Reduced: evidence demonstrating a reduced transmissibility/severity compared to the WT; No evidence: no evidence has been demonstrated.

**Additional file 2:** CNS cell expression of ACE2, TMPRSS2, NRP1 and CD147 entry factors. Cells seeded on µ-slide 8-well ibiTreat were fixed, permeabilised and stained for nuclei (blue), ACE2 (yellow), TMPRSS2 (Red), NRP1 (green) and/or CD147 (orange). Cells were then visualised by inverted microscope with a 40 × objective (405 Diode and 633 and/or 488 lasers).

**Additional file 3:** Confirmation of the infectivity of the SARS-CoV-2 viral stocks used in this study. VERO E6 cells were infected for 24 h at various MOI (0.1 – 1 – 2.5 – 5) with WT (blue), Alpha (pink), Beta (green), Delta (brown), Eta (orange) or Omicron (yellow-green) viruses. The uninfected condition for each CNS cell (grey) is represented and formed the background. The mean and SEM for 3 different donors/passages are shown. The normalised plaque area (Fluorescent viral areas (µm^2^) normalised over fluorescent nuclei areas (µm^2^)) is presented. Asterisks denote statistically significant data as defined by two-way analysis of variance (ANOVA) with corrections for multiple comparisons (Dunnett) (*P < 0.05, **P < 0.01, ***P < 0.001, ****P < 0.0001).

**Additional file 4:** Delta and Eta infections are not cytopathic for CNS cells. (A, B) astrocytes, (C, D) hCMEC/d3, (E, F) HBVP and (G, H) HMC3 were infected with Delta (A, C, E, G) or Eta (B, D, F, H) viruses at MOI 0.1 (Delta: brown; Eta: yellow) or MOI 1 (Delta: black, Eta: orange) for 6 days and the impedance was recorded in real time continuously. The mean and SEM for 3 to 5 different donors/passages are presented in percentage of the uninfected control (red).

## Data Availability

Not applicable.

## Abbreviations

ACE2: Angiotensin-converting enzyme 2
BBB: Blood–brain barrier
BSG: Basigin
CDH5: Cadherin 5
CLDN5: Claudin-5
COVID-19: Coronavirus disease 2019
CNS: Central nervous system
EMMPRIN: Extracellular matrix metalloproteinase inducer
F11R: F11 receptor
GLT-1: Glutamate transporter 1
HBVP: Human brain vascular pericytes
hCMEC/d3: Human cerebral microvascular endothelial cells
HCoV: Human coronaviruses (coronaviruses pathogenic for humans)
HMC3: Human microglial cells
MMP-9: Matrix metallopeptidase 9
MOI: Multiplicity of infection
NRP1: Neuropilin-1
OCLN: Occludin
ORF: Open reading frame
ROS: Reactive oxygen species
SARS-CoV-2: Severe acute respiratory syndrome coronavirus 2
TJP1: Tight junction protein 1
TMPRSS2: Transmembrane protease serine 2
WT: Wild-type
