# Supplementary material for: Differential effects of SARS-CoV-2 variants on central nervous system cells and blood–brain barrier functions
Source: J Neuroinflammation. 2023 Aug 3;20:184. doi: 10.1186/s12974-023-02861-3 (PMC10398935; doi:10.1186/s12974-023-02861-3)
Supplement: Supplementary file 1 — Additional file 1: Mutations and impact on transmissibility and severity of Alpha, Beta, Delta, Eta and Omicron variants. ORF: Open Reading Frame; E: Envelope; N: Nucleocapsid; M: Matrix. 1: in-house sequencing; 2: covariants.org enable by GISAID; 3: European Centre for Disease Prevention and Control. Increased: evidence demonstrating an increased transmissibility/severity compared to the WT; Reduced: evidence demonstrating a reduced transmissibility/severity compared to the WT; No evidence: no evidence has been demonstrated. [file 12974_2023_2861_MOESM1_ESM.pdf]

WHO label<br>Lineage	Emergence	Mutations				Impacts on[3]
		Spike[1]	ORF1ab[2]	Other ORFs[2]	E/M/N[2]	
**Alpha**<br>**B.1.1.7**	United Kingdom<br><br>September 2020	Δ69-70<br>Δ144<br>N501Y<br>A570D<br>D614G<br>P681H<br>T716I<br>S982A<br>D1118H	ORF1a: T1001I<br>ORF1a: A1708D<br>ORF1a: I2230T<br>ORF1a: Δ3675-3677<br>ORF1b: P314L	ORF8: Q27*<br>ORF8: R52I<br>ORF8: Y73C	N: D3L<br>N: R203K<br>N: G204R<br>N: S235F	Transmissibility: increased<br><br>Severity: increased
**Beta**<br>**B.1.351**	South Africa<br><br>October 2020	D80A<br>D215G<br>Δ241-243<br>K417N<br>E484K<br>N501Y<br>D614G<br>A701V	ORF1a: T265I<br>ORF1a: K1655N<br>ORF1a: K3353R<br>ORF1a: Δ3675-3677<br>ORF1b: P314L	ORF3a: Q57H	N: T205I<br>E: P71L	Transmissibility: increased<br><br>Severity: increased
**Delta**<br>**B.1.617.2**	India<br><br>December 2020	T19R<br>E156G<br>Δ157-158<br>L452R<br>T478K<br>D614G<br>P681R<br>D950N	ORF1b: P314L<br>ORF1b: G662S<br>ORF1b: P1000L	ORF3a: S26L<br>ORF7a: V82A<br>ORF7a: T120I<br>ORF8: Δ119-120<br>ORF9b: T60A	N: D63G<br>N: R203M<br>N: D377Y<br>M: I82T	Transmissibility: increased<br><br>Severity: increased
**Eta**<br>**B.1.525**	England, Nigeria<br><br>December 2020	Q52R<br>A67V<br>Δ69-70<br>Δ144<br>E484K<br>D614G<br>Q677H<br>F888L	ORF1a: T2007I<br>ORF1a: Δ3675-3677<br>ORF1b: P314F	ORF6: Δ2	N: Δ2<br>N: D3Y<br>N: A12G<br>N: T205I<br>E: L21F<br>M: I82T	Transmissibility: no evidence<br><br>Severity: no evidence
**Omicron**<br>**BA.1**	South Africa<br><br>November 2021	A67V<br>Δ69-70<br>T95I<br>Δ142-144<br>Y145D<br>Δ211<br>L212I<br>G339D<br>S371L<br>S373P<br>S375F<br>K417N<br>N440K<br>G446S<br>S477N<br>T478K<br>E484A<br>Q493R<br>G496S<br>Q498R<br>N501Y<br>Y505H<br>T547K<br>D614G<br>H655Y<br>N679K<br>P681H<br>A701V<br>N764K<br>D796Y<br>N856K<br>Q954H<br>N969K<br>L981F	ORF1a: K856R<br>ORF1a: Δ2083<br>ORF1a: L2084I<br>ORF1a: A2710T<br>ORF1a: T3255I<br>ORF1a: P3395H<br>ORF1a: Δ3674-3676<br>ORF1a: I3758V<br>ORF1b: P314L<br>ORF1b: I1566V	ORF9b: P10S<br>ORF9b: Δ27-29	N: P13L<br>N: Δ31-33<br>N: R203K<br>N: G204R<br>E: T9I<br>M: D3G<br>M: Q19E<br>M: A63T	Transmissibility: increased<br><br>Severity: reduced
