# Supplementary figures and images for: Differential effects of SARS-CoV-2 variants on central nervous system cells and blood–brain barrier functions
Source: J Neuroinflammation. 2023 Aug 3;20:184. doi: 10.1186/s12974-023-02861-3 (PMC10398935; doi:10.1186/s12974-023-02861-3)

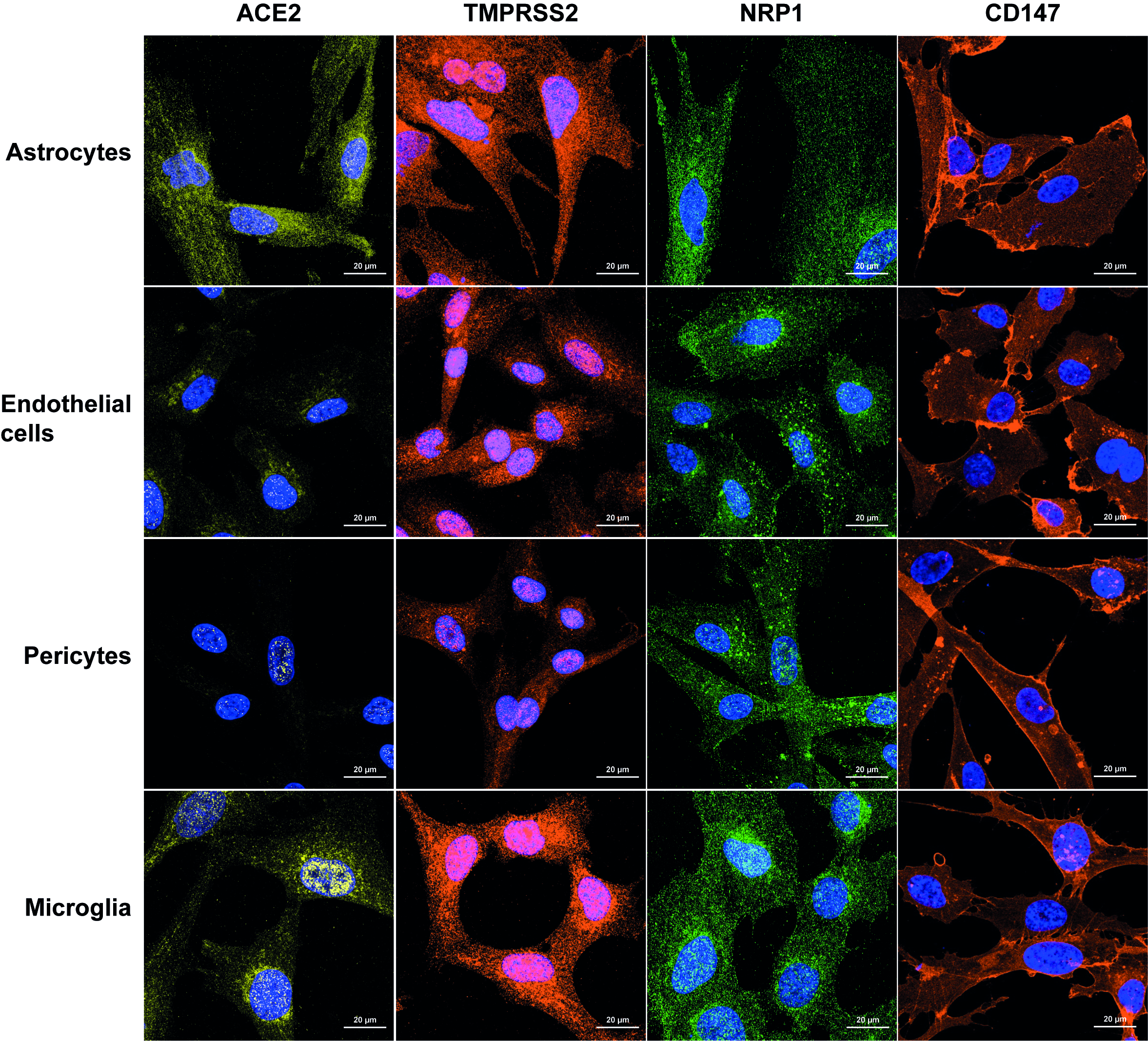

Supplement: Supplementary file 2 — Additional file 2: CNS cell expression of ACE2, TMPRSS2, NRP1 and CD147 entry factors. Cells seeded on µ-slide 8-well ibiTreat were fixed, permeabilised and stained for nuclei (blue), ACE2 (yellow), TMPRSS2 (Red), NRP1 (green) and/or CD147 (orange). Cells were then visualised by inverted microscope with a 40 × objective (405 Diode and 633 and/or 488 lasers). [file 12974_2023_2861_MOESM2_ESM.tif]

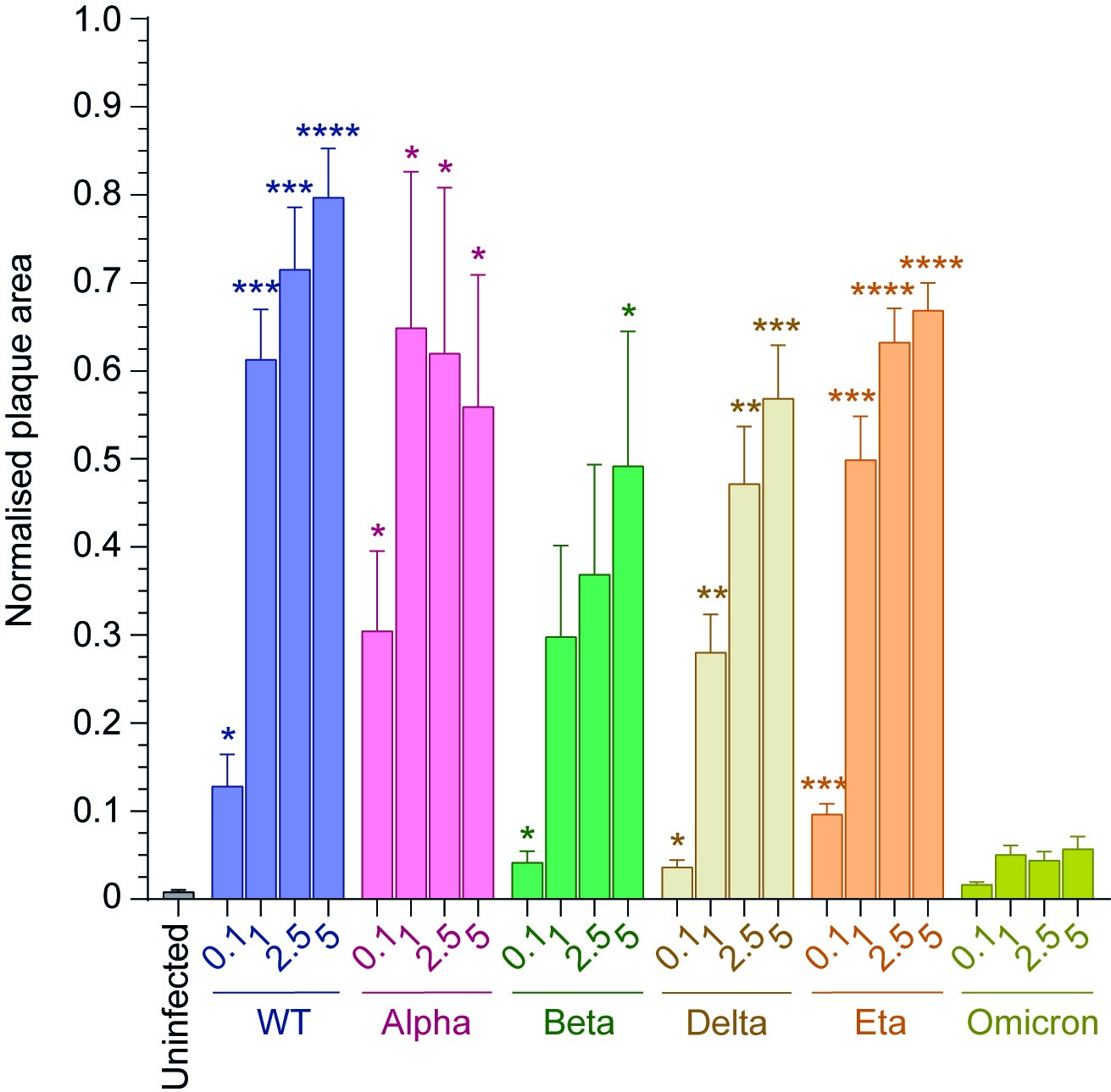

Supplement: Supplementary file 3 — Additional file 3: Confirmation of the infectivity of the SARS-CoV-2 viral stocks used in this study. VERO E6 cells were infected for 24 h at various MOI (0.1 – 1 – 2.5 – 5) with WT (blue), Alpha (pink), Beta (green), Delta (brown), Eta (orange) or Omicron (yellow-green) viruses. The uninfected condition for each CNS cell (grey) is represented and formed the background. The mean and SEM for 3 different donors/passages are shown. The normalised plaque area (Fluorescent viral areas (µm2) normalised over fluorescent nuclei areas (µm2)) is presented. Asterisks denote statistically significant data as defined by two-way analysis of variance (ANOVA) with corrections for multiple comparisons (Dunnett) (*P < 0.05, **P < 0.01, ***P < 0.001, ****P < 0.0001). [file 12974_2023_2861_MOESM3_ESM.tif]

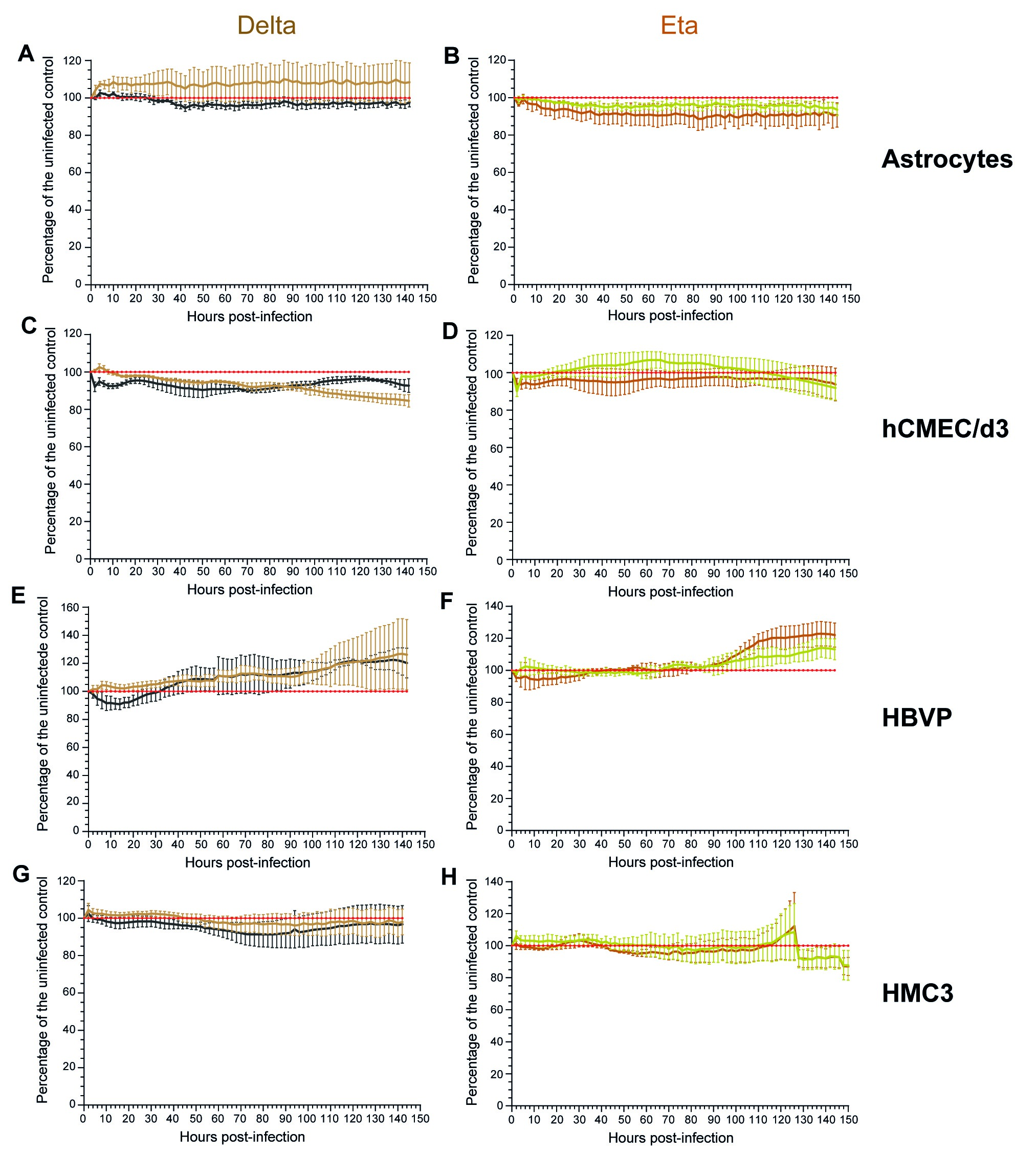

Supplement: Supplementary file 4 — Additional file 4: Delta and Eta infections are not cytopathic for CNS cells. (A, B) astrocytes, (C, D) hCMEC/d3, (E, F) HBVP and (G, H) HMC3 were infected with Delta (A, C, E, G) or Eta (B, D, F, H) viruses at MOI 0.1 (Delta: brown; Eta: yellow) or MOI 1 (Delta: black, Eta: orange) for 6 days and the impedance was recorded in real time continuously. The mean and SEM for 3 to 5 different donors/passages are presented in percentage of the uninfected control (red). [file 12974_2023_2861_MOESM4_ESM.tif]
